# Multicenter case–control study protocol of pneumonia etiology in children: Global Approach to Biological Research, Infectious diseases and Epidemics in Low-income countries (GABRIEL network)

**DOI:** 10.1186/s12879-014-0635-8

**Published:** 2014-12-10

**Authors:** Valentina Sanchez Picot, Thomas Bénet, Melina Messaoudi, Jean-Noël Telles, Monidarin Chou, Tekchheng Eap, Jianwei Wang, Kunling Shen, Jean-William Pape, Vanessa Rouzier, Shally Awasthi, Nitin Pandey, Ashish Bavdekar, Sonali Sanghvi, Annick Robinson, Bénédicte Contamin, Jonathan Hoffmann, Maryam Sylla, Souleymane Diallo, Pagbajabyn Nymadawa, Budragchaagiin Dash-Yandag, Graciela Russomando, Wilma Basualdo, Marilda M Siqueira, Patricia Barreto, Florence Komurian-Pradel, Guy Vernet, Hubert Endtz, Philippe Vanhems, Gláucia Paranhos-Baccalà

**Affiliations:** Emerging Pathogens Laboratory – Fondation Mérieux, Centre International de Recherche en Infectiologie (CIRI) Inserm U1111, CNRS UMR5308, ENS de Lyon, UCBL1, 21, Avenue Tony Garnier, Lyon, 69007 France; Infection Control and Epidemiology Unit, Edouard Herriot Hospital, Hospices Civils de Lyon, Lyon, France; Epidemiology and Public Health Unit, University of Lyon 1, Lyon, France; Faculty of Pharmacy, University of Health Sciences, Phnom Penh, Cambodia; Department of Pneumology, National Pediatric Hospital, Phnom Penh, Cambodia; MOH Key Laboratory of Systems Biology of Pathogens and Dr. Christophe Mérieux Laboratory, IPB, CAMS-Fondation Mérieux, Institute of Pathogen Biology (IPB), Chinese Academy of Medical Sciences (CAMS) & Peking Union Medical College), Beijing, China; Key Laboratory of Major Diseases in Children and National Key Discipline of Pediatrics (Capital Medical University), Ministry of Education, Beijing Pediatric Research Institute, Beijing Children’s Hospital, Capital Medical University, Beijing, China; GHESKIO (Groupe Haïtien d’Etude du Sarcome de Kaposi et des Infections Opportunistes) Centers, Port au Prince, Haiti; Chatrapati Shahuji Maharaj University, Lucknow, India; KEM Hospital Research Center, Pune, India; Hôpital Femme-Mère-Enfant, Antananarivo, Madagascar; Fondation Mérieux, Centre d’Infectiologie Charles Mérieux (CICM), Antananarivo, Madagascar; Gabriel Touré Hospital, Bamako, Mali; Centre d’Infectiologie Charles Mérieux (CICM), Bamako, Mali; Mongolian Academy of Medical Sciences, Ulaanbaatar, Mongolia; Bayanzurkh District General Hospital, Ulaanbaatar, Mongolia; Research Institute of Health, Asuncion, Paraguay; Hospital Pediátrico “Niños de Acosta Ñu”, San Lorenzo, Paraguay; Respiratory virus Laboratory, Oswaldo Cruz Foundation, Hospital Bonsucesso, Rio de Janeiro, Brazil; the pneumonia GABRIEL network, Brazil

**Keywords:** Pneumonia, Children, Case–control, Etiology, Developing and emerging countries

## Abstract

**Background:**

Data on the etiologies of pneumonia among children are inadequate, especially in developing countries. The principal objective is to undertake a multicenter incident case–control study of <5-year-old children hospitalized with pneumonia in developing and emerging countries, aiming to identify the causative agents involved in pneumonia while assessing individual and microbial factors associated with the risk of severe pneumonia.

**Methods/design:**

A multicenter case–control study, based on the GABRIEL network, is ongoing. Ten study sites are located in 9 countries over 3 continents: Brazil, Cambodia, China, Haiti, India, Madagascar, Mali, Mongolia, and Paraguay. At least 1,000 incident cases and 1,000 controls will be enrolled and matched for age and date. Cases are hospitalized children <5 years with radiologically confirmed pneumonia, and the controls are children without any features suggestive of pneumonia. Respiratory specimens are collected from all enrolled subjects to identify 19 viruses and 5 bacteria. Whole blood from pneumonia cases is being tested for 3 major bacteria. *S. pneumoniae*-positive specimens are serotyped. Urine samples from cases only are tested for detection of antimicrobial activity. The association between procalcitonin, C-reactive protein and pathogens is being evaluated. A discovery platform will enable pathogen identification in undiagnosed samples.

**Discussion:**

This multicenter study will provide descriptive results for better understanding of pathogens responsible for pneumonia among children in developing countries. The identification of determinants related to microorganisms associated with pneumonia and its severity should facilitate treatment and prevention.

**Electronic supplementary material:**

The online version of this article (doi:10.1186/s12879-014-0635-8) contains supplementary material, which is available to authorized users.

## Background

**Figure 1:**
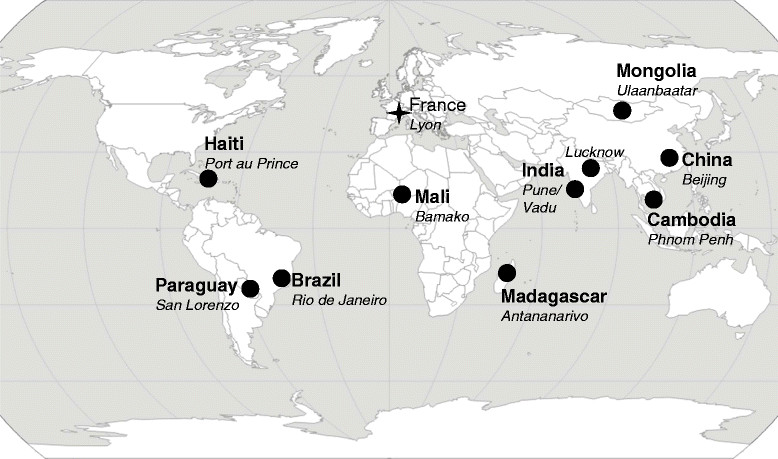
**Map of the world showing participating sites of the multicenter case–control study of pneumonia etiology in children.**

With more than 120,000 million episodes occurring worldwide every year [1], pneumonia is the leading cause of child mortality from infectious diseases, accounting for an estimated 1 million deaths annually, and mainly afflicting children in developing countries according to Global Health Observatory [2]. Mortality attributed to pneumonia has decreased since 2000, but remains a major public health concern [3]. Improvements in medical care and the implementation of vaccination policies may have contributed to the decline in mortality [1]. However, data on the etiology and epidemiology of pneumonia in developing countries are still insufficient. Indeed, large studies that investigated the etiology of pneumonia in children were undertaken more than 20 years ago [4]. During this time, several epidemiological factors, such as human immunodeficiency virus (HIV) pandemic, *Streptococcus pneumoniae* and *Haemophilus influenzae* vaccine implementation, might have modified the global distribution of causative pathogens [1],[5],[6]. Moreover, the development of molecular techniques in the last few decades has led to the detection of numerous pathogens, mainly viruses [7],[8] (Figure 1).

The etiology of pneumonia could be either bacterial or viral.[9] The main known causative pathogens are *Streptococcus pneumoniae* [5],[10]. *Haemophilus influenzae* [6], and respiratory syncitial virus (RSV) [11]. Interactions between viruses, bacteria and host are complex and remain poorly explored [12],[13]. In outbreak situations, data on the spatial and temporal distribution of etiological agents are key to identification and control. Several predictors of pneumonia or severe pneumonia have been found [14],[15]. However, the association with pneumonia etiology has not been fully investigated [15]. Indeed, the identification of factors strongly linked with complications or death could help in the development of appropriate therapeutic management strategies for these patients.

The purpose of the present work is to summarize the protocol of the multicenter incident case–control study of pneumonia in children less than 5 years of age, aiming to identify the etiological agents and related determinants involved in pneumonia. The countries participating in this study account for a significant part of the pneumonia burden in children worldwide. For example, India recorded around 35 million new cases of pneumonia in 2010, with the highest numbers of deaths in the world (390,000), while China has 6 million cases and ranks fifth, with about 43,000 deaths annually [16]. Although pneumonia is still a major cause of mortality in other countries, observational data on pneumonia etiology are very sparse [16]. In Madagascar, for example, apart from earlier studies on influenza A and B viruses [17], to our knowledge, no other data are available on respiratory viruses and their transmission at the community level.

## Methods/Design

### Study objectives

The study’s primary objectives are to identify the causative agents involved in the onset of pneumonia in children and to assess individual and microbial factors associated with the risk of pneumonia, including severe pneumonia. Its secondary objectives are: 1) to examine the value of C-reactive protein (CRP) and procalcitonin (PCT) in predicting the severity of pneumonia or infection by particular pathogens, 2) to estimate the involvement of etiological agents in pneumonia, and 3) to characterize new infectious agents or variants in pneumonia of unknown etiology.

### Study sites and design

This prospective, hospital-based, multicenter case–control study is being conducted at 10 sites in 9 countries – Brazil, Cambodia, China, Haiti, India (2 sites: Pune/Vadu and Lucknow), Madagascar, Mali, Mongolia, Paraguay – on 3 continents. Enrollment of at least 100 incident cases and 100 controls during 12 months is planned for each participating site for a total sample of at least 2,000 subjects. The participating sites are members of the GABRIEL network of Fondation Mérieux [18]. Table 1 reports the demographic characteristics and burden of pneumonia in each participating country. Laboratory data are generated by local sites after reaching capacity, via technology transfer and training by the Emerging Pathogens Laboratory of Fondation Mérieux (Lyon, France).

**Table 1:** **Description of sites in multicenter case–control study of pneumonia etiology in children**

Study site characteristics						Country characteristics					Introduction into national immunization program ^a^	
Hospital	Country	Town	Related research center	Population type	Climate	Country population, millions (year) ^a^	Population living in poverty, % (year) ^a^	Child mortality rate (‰) ^b^	Annual pneumonia cases ^c^	Deaths caused by pneumonia annually ^c^	PCV ^d^	Hib ^e^vaccine
General Bonsucesso Hospital	Brazil	Rio de Janeiro	Fundaçao Oswaldo Cruz	Urban	Rain/Dry	199 (2009)	6.1 (2009)	16	1,497,706	3,079	2010	1999
National Pediatric Hospital	Cambodia	Phnom Penh	Phnom Pehn Faculty of Pharmacy and University of Health Sciences	Urban	Rain/Dry	14.9 (2012)	18.6 (2009)	43	373,583	2,101	Not introduced	2010
Beijing Children’s Hospital	China	Beijing	Chinese Academy of Medical Sciences	Urban	Four seasons	1,351 (2012)	11.8 (2009)	15	6,488,544	43,089	Not introduced	Not introduced
Tent City Hospital^f^	Haïti	Port au Prince	GHESKIO Centers	Urban/rural	Rain/Dry	10.2 (2012)	61.7 (2001)	70	345,081	4,090	Not introduced	2012
KEM Hospital Research Center Shirdi Sai Baba Rural Hospital	India	Pune/Vadu	KEM Hospital Research Center Sai Baba Rural Hospital	Urban/rural	Three seasons	1,236.7 (2012)	32.7 (2010)	61	35,361,230	388,144	Not introduced	2011
Chatrapati Shahuji Maharaj University Hospital	India	Lucknow	Chatrapati Shahuji Maharaj University	Urban/rural	Rain/Dry	1,236.7 (2012)	32.7 (2010)	61	35,361,230	388,144	Not introduced	2011
Hôpital Femme-Mere-Enfant	Madagascar	Antananarivo	Centre Charles Mérieux	Urban	Rain/Dry	22.3 (2012)	81.3 (2010)	62	1,051,407	2,638	2011	2008
Gabriel Toure Hospital	Mali	Bamako	Centre Charles Mérieux	Urban/rural	Rain/Dry	14.9 (2012)	50.4 (2010)	176	932,894	7,893	2008	2011
Bayanzurkh District General Hospital	Mongolia	Ulaanbaatar	Mongolian Academy of Medical Sciences	Urban	Four seasons	2.8 (2012)	27.4 (2012)	31	60,292	332	Not introduced	2008
Pediatric General Hospital, Niños de Acosta Ñu	Paraguay	San Lorenzo	Research Institute of Health	Urban/rural	Rain/Dry	6.7 (2009)	7.2 (2012)	22	139,661	368	2012	2002

^a^International Vaccine Access Center (IVAC), Johns Hopkins Bloomberg School of Public Health. Vaccine Information Management System (VIMS) Global Vaccine Introduction Report, October 2013. www.jhsph.edu/ivac/vims.html.

^b^World Bank Group: http://povertydata.worldbank.org.

^c^Data from Rudan I, O’Brien KL, Nair H, et al. Epidemiology and etiology of childhood pneumonia in 2010: estimates of incidence, severe morbidity, mortality, underlying risk factors and causative pathogens for 192 countries. *J Glob Health* 2013;3:010401.

^d^Pneumococcal conjugate vaccine.

^e^ *Haemophilus influenzae* type B.

^f^The hospital is settled in a tent city community.

### Study participants

The study population, comprised of children under 5 years of age, complies with protocol definitions and inclusion criteria. Eligible patients are identified by study pediatricians at each participating site. Cases are defined by the following criteria: 1) Hospitalized patients aged between 2 months and 59 months, 2) Clinical features of pneumonia, as described below, 3) radiological confirmation of pneumonia on chest X-ray as per WHO guidelines [19], and 3) Informed consent statement signed by the children’s parents or legal guardian. The exclusion criterion for cases are the following: presence of wheezing at auscultation, or minors whose parents or legal guardian declined to sign the informed consent statement.

Controls are defined by the following criteria: 1) Patients aged between 2 months and 59 months, 2) Hospitalized for surgery or in a routine outpatient practice environment, and 3) Informed consent statement signed by the children’s parents or legal guardian. Exclusion criteria for the controls are: 1) Any symptom suggestive of respiratory illness, or 2) Minors whose parents or legal guardian decline to sign the informed consent statement. Cases and controls are matched for study site, age (±1 year) and calendar date of hospital admission (±1 month).

### Definition of pneumonia

Pneumonia cases are defined by the following:

- Cough and/or dyspnea, and
- Tachypnea, as defined by the World Health Organization (WHO) (in children between 2 and 12 months of age: breathing rate ≥50 cycles per minute; in children between 12 months and 59 months of age: breathing rate ≥40 cycles per minute) [20], and
- Absence of wheezing at auscultation, and
- First symptoms appearing within the last 14 days, and
- Radiological confirmation of pneumonia as per WHO guidelines [19].

### Data sources and quality control

Research conduct and management are organized by the Emerging Pathogens Laboratory of Fondation Mérieux, primarily for evaluation and monitoring of the ethics, regulatory, clinical, laboratory and data generation components at each participating site. Tasks and responsibilities are based on standard research guidelines, including standard operating procedures. Data quality is monitored and evaluated by each site and by the Emerging Pathogens Laboratory for pooled data analysis. Demographic characteristics, underlying diseases, medical history, clinical examination at enrollment, radiological findings, vaccinations, and outcome are recorded prospectively for each patient on a standardized form (Table 2).

**Table 2:** **Individual questionnaire among cases and controls in multicenter case–control study of pneumonia etiology in children**

Category	Information	
General	Hospital	Country
Demographic	Gender	Date of birth
Underlying diseases	Heart disease	Lung disease
	Tuberculosis	Asthma
	Pneumonia	HIV infection
	Others	
Medical history	Contact with tuberculosis	Previous influenza
	Prior treatment with antibiotics	Prior treatment for fever
	Date of first symptoms	
Clinical examination at enrollment	Weight	Height
	Arm circumference	Temperature
	Blood pressure	Breathing rate
	Arterial oxygen saturation	Cough
	Dyspnea	Lower chest indrawing
	Cyanosis	Pulmonary crackles
	Dullness to percussion of the thorax	Rhonchi
	Wheezing	Rasping
	Diminished breathing sounds	Convulsions
	Other pulmonary signs and symptoms	Rhinopharyngitis
	Prostration or lethargy	Conjunctivitis
	Otitis	Inability to drink
	Skin rash	Diarrhea
	Vomiting	Other signs
Radiological	Opacification	Abscess
findings	Interstitial syndrome	Pneumothorax
(cases only)	Alveolar infiltrate	Parenchymatous infiltrate
Vaccination (dates, number of doses)	Pneumococcal conjugate vaccine Influenza vaccine	Diphtheria, tetanus, pertussis, hepatitis B, and *Haemophilus influenzae* type B vaccine
During hospital stay	Antibiotics (dates, doses, duration)	Oxygen therapy
	Other drugs	Death
	Acute complications	Cause of death
	Recovery	

Data quality reporting is redacted for each site to ensure the conformity of all study data variables, it will detailed enrollment and case definition conformity, errors, and missing data [21],[22]. This process will be applied to data analysis of each enrolled case and control. Each potential error is discussed, and a final rule applied. The principal investigator at each site is informed for queries regarding this quality assessment and is involved in resolution. A tracking system is applied to the process.

### Biological samples

Samples are collected in the first 48 hours of the patient hospitalization. Nasal swabs/aspirates are collected from all pneumonia cases and controls. This collection procedure is performed by trained clinical staff, nurses and the principal investigator. Additional specimens are from cases only, including urine, whole blood and pleural effusions (Table 3). Antibiotic substances are detected in urine samples from all pneumonia cases to detect antibiotic usage history. Whole blood is collected and distributed in Ethylene-diamine-tetra-acetic acid (EDTA) vials for preservation and in dry tubes for serum specimens. EDTA-whole blood samples allow complete blood count, blood culture and real-time multiplex polymerase chain reaction (PCR) assay for the identification of *Staphylococcus aureus*, *Streptococcus pneumoniae* and *Haemophilus influenzae* type B. CRP and PCT are measured quantitatively in serum. Respiratory specimens (nasal swabs/aspirates and pleural effusions) will permit to identify viruses and bacteria by real-time multiplex PCR assay with a panel of 19 viruses and 5 bacteria (FTD respiratory pathogens 21 PLUS (Fast-track Diagnostic, Luxemburg). The pathogens targeted are: influenza A, H1N1, influenza B, coronavirus 229E, coronavirus OC43, coronavirus NL63, coronavirus HKU1, parainfluenza virus 1, parainfluenza virus 2, parainfluenza virus 3, parainfluenza virus 4, human metapneumoviruses A and B, rhinovirus, RSVs A and B, adenovirus, enterovirus, parechovirus, bocavirus), *Mycoplasma pneumoniae*, *Chlamydia pneumoniae*, *S. aureus*, *S. pneumoniae*, and *H. influenzae* type B. *S. pneumoniae*-positive specimens are serotyped with a multiplex real-time PCR method that detects 29 different serotypes (1, 3, 4, 5, 6A/B, 7C, 7F, 8, 9V, 10A, 11A, 12F, 14, 15A, 15B/C, 16F, 17F, Sg18C, 19A, 19F, 20, 22F, 23F, 31, 33F, 34, 35B, 35F, 38 and Lyt A). A centralized, blinded PCR respiratory quality control panel is also provided to all sites to ensure procedure validation on site before specimens are processed locally. The control panel is processed every 3 months at each laboratory site. Each laboratory has to successfully test the control panels to proceed with the study.

**Table 3:** **Laboratory tests in multicenter case–control study of pneumonia etiology in children**

Samples	Population	Analyses
Nasal swab/aspirate	Cases and controls	Molecular detection of viruses and bacteria *S. pneumoniae* typing
Pleural effusion	Cases (if pleuritis)	Molecular detection of viruses and bacteria *S. pneumoniae* typing
Whole blood	Cases	Molecular detection of viruses and bacteria
		*S. pneumoniae* typing
		Blood culture
		Blood count
Serum	Cases	C-reactive protein
		Procalcitonin
Urine	Cases	Broad antibiotic detection

### Potential bias

Because the study concerns hospitalized pneumonia cases, the magnitude of putative selection bias according to disease severity will be evaluated for cases and controls. Control charts will detect evolving selection bias over time at particular sites [21]. Patients’ characteristics and the clinical presentation of pneumonia cases will be described and compared between sites. Precise evaluation of diagnostic criteria and standardized biological tests with central analysis should reduce misclassification bias. A flow chart describing the quality of case enrollment and follow-up is constructed by site and for pooled populations. Based on past experiences in multicenter pneumonia studies, particular attention will be paid to standardization of case definition [4],[23]. Moreover, heterogeneity of the raw data will be compared between sites. This process will precisely define the population that is enrolled in the pooled analysis, according to data validity and population homogeneity. Multivariate analysis (described above) will take the main confounding factors into account.

### Study size power calculation

With 100 cases and 100 controls per site, analysis in a particular site will have 80% power to detect odds ratios (OR) ≥3 with prevalence of exposure ≥20% for the controls, whatever the prevalence of exposure associated with a case. With 1,000 cases and 1,000 controls, overall analysis will have 90% power to detect OR ≥ 2 with exposure in controls ≥5%, whatever the prevalence of exposure associated with a case. Therefore, the study will include at least 2,000 patients; at least 100 cases and 100 controls enrolled at each participating site.

### Statistical methods

An anonymous database will be built and analyzed. Quantitative variables will be described and categorized according to their distribution in the study population. The proportion of missing data will be described for all variables, and the mechanism of missing data will be researched; a multiple imputation process of missing data will be implemented, if necessary. Descriptive analysis will address each covariate for the entire population, and stratified by site. If multiple microorganisms are detected, typology of co-infections will be developed. Co-infections/colonization will be reported by site, patients’ characteristics and time.

Patients’ characteristics and microbiological data on cases and controls will be compared. Mixed effects logistic univariate and multivariate regression modeling will be applied to assess factors associated with pneumonia or infection by particular pathogens. These models will take into account heterogeneity in baseline risk between sites and countries as well as the nested nature of the data. Indeed, microbial agents are nested in patients because they could be infected by several agents; patients are nested in sites and countries. Other analysis will focus on factors associated with co-infection, for each possible pair or trio of microorganisms. Individual and microbial factors linked with the risk of severe disease or death will be analyzed by survival models, if applicable. Sensitivity, specificity and predictive values will be calculated to research the value of CRP and PCT in predicting the severity of pneumonia or infection by particular pathogens. Complementary methods and stratified analyses will be deployed, if necessary, to study the distribution of the causative agents of pneumonia according the underlying medical condition such as HIV for example.

### Ethics and confidentiality

The study protocol, informed consent statement, clinical research form, any amendments and all other study documents have been submitted to and approved by the institutional ethics committee of each site: Brazil (*Conselho Nacional de Saúde, Comissão Nacional de Ética em Pesquisa, Ministerio da Saúde*), China (National Ethics Committee, Ministry of Health, Cambodia (National Ethics Commitee for Health Research, Ministry of Health), Haïti (*Comité des Droits Humains des Centres GHESKIO, Comité National d’Ethique, Ministère de la Santé Publique et de la Population*, and Institutional Review Board of Weill Cornell Medical College), India Lucknow center (Institutional Ethics Committee, Chhatrapati Shahuji Maharaj Medical University, Uttar Pradesh), India Pune (Ethics Commity at KEM hospital research centre), Madagascar (*Comité d’Ethique, Ministère de la Santé Publique*), Mali (*Comité national d’éthique pour la santé et les sciences de la vie*), Mongolia (Ethical committee for medical research), and Paraguay (*Comité de Ética en Investigación del Instituto de Investigaciones en Ciencias de la Salud de la Universidad Nacional de Asunción*).

## Discussion

Multicenter pneumonia study analysis will be reported for publication in 2015. Individual site analysis, focusing on particular interests, will also be described and published at a later date.

Study analysis will generally focus on the description of causative pathogens at the onset of pneumonia for the entire population and by country site. Several studies have already investigated the etiology of pneumonia [24]. However, they were limited by either unicentric study design [14], focused on a particular pathogen [25], lacked a control group [26], or employed diagnosis techniques with limited sensitivity [24]. Many reports came from high-income countries, in highly-selected populations, such as patients with pneumonia requiring intensive care unit stay [27]. Data are lacking on pneumonia etiology in developing countries based on worldwide distribution of study sites. A better understanding of pathogens causing pneumonia in children should contribute to improved preventive – through implementation of vaccine policies – and therapeutic management, ultimately leading to reduced morbidity and mortality of pneumonia in children. Moreover, CRP combined with PCT has demonstrated value in predicting pneumonia in adults, but interest in pediatric populations as not been well demonstrated [28],[29].

Thus, our study should provide new data on the following questions: What are the viral and bacterial etiological agents of pneumonia in children under 5 years of age in developing and emerging countries? What is their seasonal distribution? What are the circulating pneumococcal serotypes? What is the distribution of co-infections? What is the clinical profile of co-infected patients? Are CRP and PCT predictive markers of pneumonia etiology? Which pathogen is presented in samples without any etiology?

Several study weaknesses should be considered, including bias, confounders and statistical power. Firstly, potential selection bias with different mechanisms of selection between sites might occur. Indeed, a particular site might include cases according to a definition that does not exactly conform to standardized case definition, as with the controls. Another example might be the selection of subjects due to local clinical practices or field experiences. These different mechanisms of selection could jeopardize data interpretation, by site and pooled analysis [30]. Some subjects’ results might be excluded from the overall analysis. Secondly, external validity could be questioned by site compared to the country population to assess representativeness of the study population, and compared with the world population for pooled analysis [30]. Thirdly, limited information might be available on exposures preceding pneumonia occurrence. Also, some confounding related to nutrition or parental history factors, will be missing at the individual level. However, the geographical (i.e., rainfall, temperature) and socio-cultural (i.e., type of population) context as well as study site and local health care system characteristics will be described [31]. Finally, the quality and distribution of variables will limit some statistical analyses, with an impact on study power. Thus, multiple imputations as assumption checking before modeling will also be a key issue before running statistical analysis.

The study’s main strength is the prospective collection of multiple biological samples that should permit the diagnosis of etiological agents involved in pneumonia as well as the importance of colonization by other microbial agents. Another advantage is the multicenter study design that will permit the description of various etiologies of pneumonia in study-related regions of the world [18]. It is noteworthy that the two most populous countries in the world, which represent a large burden of pneumonia incidence and mortality worldwide – China and India – are part of the study [16]. The results of our multicenter pneumonia study could be possibly compared with those of other contemporary pneumonia investigations. In this regard, the ongoing Pneumonia Etiology Research for Child Health (PERCH) study will be particularly informative [32]. Indeed, study design, case definition, molecular techniques and biological samples, while not identical, are not very different between the two multicenter works [33],[34]. PERCH is larger than the present study, but countries such as India and China are not participating in it while they are included in our multicenter pneumonia investigation. Also, comparative analyses might be considered as a perspective that could offer new insights into pneumonia etiologies worldwide.

## Conclusion

In conclusion, our multicenter pneumonia study will detail pneumonia etiologies among children in 9 countries on 3 continents. The results could be helpful not only in improving individual care but also in formulating appropriate public health policies [35].

## Authors’ original submitted files for images

Below are the links to the authors’ original submitted files for images. Authors’ original file for figure 1

## Abbreviations

CRP: C-reactive protein
EDTA: Ethylene-diamine-tetra-acetic acid
GABRIEL: Global Approach to Biological Research on infectious, Epidemics in Low-income countries
HIV: Human immunodeficiency virus
OR: Odds ratio
PERCH: Pneumonia Etiology Research for Child Health
PCR: polymerase chain reaction
PCT: Procalcitonin
RSV: Respiratory syncytial virus
WHO: World Health Organization
