# Supplementary figures and images for: Multicenter case–control study protocol of pneumonia etiology in children: Global Approach to Biological Research, Infectious diseases and Epidemics in Low-income countries (GABRIEL network)
Source: BMC Infect Dis. 2014 Dec 10;14:635. doi: 10.1186/s12879-014-0635-8 (PMC4272811; doi:10.1186/s12879-014-0635-8)

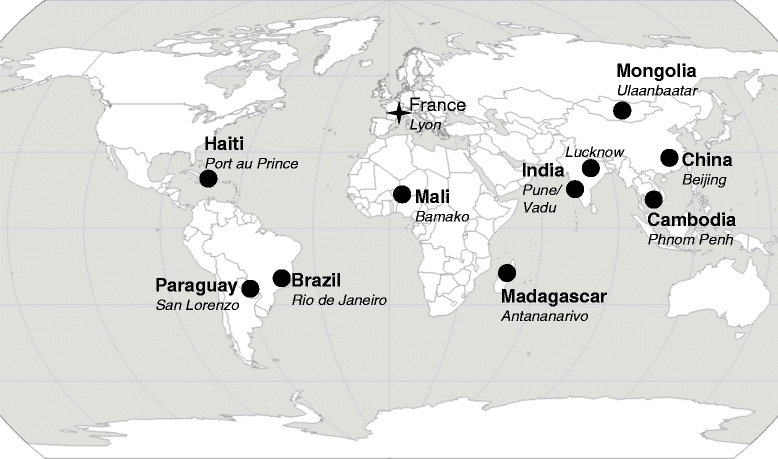

Supplement: Supplementary file 1 — Authors’ original file for figure 1 [file 12879_2014_635_MOESM1_ESM.gif]
